# Developing and Testing a Protocol for Managing Cardiopulmonary Resuscitation of Patients with Suspected or Confirmed COVID-19: In Situ Simulation Study

**DOI:** 10.2196/38044

**Published:** 2022-06-16

**Authors:** Azizeh Sowan, Jenny Heins, Christopher Dayton, Elizabeth Scherer, Wing Sun Tam, Haritha Saikumar

**Affiliations:** 1 School of Nursing The University of Texas Health at San Antonio San Antonio, TX United States; 2 Center for Clinical Excellence University Health San Antonio, TX United States; 3 Division of Pulmonary and Critical Care Medicine Department of Emergency Medicine The University of Texas Health at San Antonio San Antonio, TX United States; 4 Division of Trauma and Emergency Surgery Department of Surgery The University of Texas Health at San Antonio San Antonio, TX United States; 5 Emergency Department Audie L Murphy Veterans Affairs Medical Center South Texas Veterans Health Care System San Antonio, TX United States; 6 Pulmonary and Critical Care The University of Texas at Austin Dell Medical School Austin, TX United States

**Keywords:** in situ simulation, critical care, COVID, cardiopulmonary, resuscitation, COVID-19, treatment, health care, nursing, health care equipment, health care resources, health care training, health care staff

## Abstract

**Background:**

Resuscitating patients with suspected or confirmed COVID-19 imposes unique challenges to organizations and code blue teams. Studies that applied the American Heart Association (AHA) COVID-19–related Interim Resuscitation Guideline and similar European guidelines are scarce.

**Objective:**

This study aimed to develop and test a cardiopulmonary resuscitation protocol based on the AHA COVID-19–related Interim Resuscitation Guideline.

**Methods:**

The study was conducted as an in situ simulation in a medical intensive care unit. The COVID-19 cardiopulmonary resuscitation protocol was created and validated by 11 health care team members and tested using 4 simulation sessions where 46 code blue team members participated. During the simulation, we observed role clarity, the effectiveness of communication, team dynamics, infection control measures, and the availability of essential supplies and equipment.

**Results:**

The main issues identified in each simulation session were debriefed to the code blue teams and used to further revise the protocol. These include the assignment of tasks, availability of equipment and supplies, and failure of communication between the in-room and out-of-room teams. Solutions included changes in the placement of team members and roles and responsibilities; the creation of an isolation code medication package, a respiratory therapy kit, and an isolation code blue bag; and the use of two-way radios and N-95 masks with eye goggles to enhance communication between the teams.

**Conclusions:**

This study shed light on the challenges to implement the AHA COVID-19–related Interim Resuscitation Guideline. The in situ simulation was an effective approach for rapid training, identifying unreliable equipment and ineffective and inefficient workflow, and managing the complexity of the physical environment.

## Introduction

The provision of cardiopulmonary resuscitation (CPR) to patients with suspected or confirmed COVID-19 presents infection control challenges inherent to the urgency of the procedure, interventions that cause aerosolization, and the need for multiple health care team members in close proximity. The additional complexity and resource management that accompany enhanced-isolation CPR (EI-CPR) also presents challenges related to communication barriers and the need to limit personnel and equipment exposure integral to safety-focused strict isolation protocols. To provide effective and safe EI-CPR, it is crucial that the risk of COVID-19 transmission to the health care team and patients be minimized and that communication, workflow, and resources be effectively managed.

In light of these challenges, the American Heart Association (AHA), in collaboration with other professional societies, has recommended modifications to the standard CPR algorithms [1] and issued a COVID-19–related Interim Resuscitation Guideline (thereafter referred to as the AHA COVID-19 Resuscitation Guideline) [2]. Studies that applied the AHA COVID-19 Resuscitation Guideline and similar European guidelines are scarce [3-7]. In response to these gaps, our simulation-based study aimed to develop and test a protocol based on the AHA COVID-19 Resuscitation Guideline for adult patients in hospital settings.

Careful integration of the AHA COVID-19 Resuscitation Guideline into the code blue workflow and testing different case scenarios based on institutional policies and available resources are crucial to the efficacy, efficiency, and safety of CPR. For example, Foong et al [5], Cheruku et al [6], and Sliver et al [7] proposed 3 different workflow models depicting the placement of code blue teams and equipment and used different communication tools between the in-room (inside the patient room) and out-of-room teams to maximize infection control measures. According to Foong et al [5], some of the unanticipated problems experienced in the new workflow were related to infection control, the conduct of resuscitation, resources, and poor communication between the teams. Continuous practice modifications and performance debriefing were necessary to optimize workflow, increase team competence, and improve the effectiveness of CPR [5].

Recent studies supported the effect of “in situ” simulation as a new simulation modality, in comparison to “off-site” simulation, on increasing confidence, competence, and teamwork and identifying system-based challenges to CPR [8,9]. Unlike off-site simulation that occurs in controlled lab environments, in situ simulation is encountered in a real-life setting where the clinical procedure occurs. The use of iterative in situ simulation to test protocols and train the multidisciplinary team members in preparation for EI-CPR for patients with suspected or confirmed COVID-19 has not been well-reported in the literature. In this in situ simulation–based study, we integrated the new AHA COVID-19 Resuscitation Guideline in our EI-CPR procedure by developing a protocol disseminated via a pocket card for the code blue team to manage the steps, roles, communication methods, and process of resuscitation.

## Methods

### Design, Setting, and Sample

This prospective observational study was conducted as an in situ simulation in a medical intensive care unit (ICU) in a 670-bed, Level I trauma magnet facility located in Southwestern United States after Institutional Review Board approval. The COVID-19 CPR protocol was created and validated by 11 health care team members. The protocol was then revised and tested using 4 simulation sessions as described below. A total of 46 health care team members participated in the 4 simulation sessions. Each mock code included 11 to 12 participants. The main eligibility criterion to participate in the in situ simulation sessions was being a member of the code blue team. Membership in code blue teams and the roles and responsibilities of the health care team members were assigned by the Directors and Patient Care Coordinators of all units in the hospital at the beginning of each shift. Non–code blue team members were not eligible to participate in the study.

Using a convenience sampling approach, our plan was to have as many code blue teams as required to streamline and refine the resuscitation process based on the new protocol (ie, until no major issues in communication, team dynamics, or equipment were found). Streamlining the process required 4 code blue teams as described below.

### Creating and Testing the New Protocol

The code blue procedure for patients with suspected or confirmed COVID-19 was discussed in the Hospital Resuscitation Services Committee meeting where the AHA COVID-19 Resuscitation Guideline was reviewed. A protocol for COVID-19 CPR was created based on the new AHA guideline by WST and HS, 2 physicians and authors of this manuscript. Key information in the protocol was also made available in a double-sided pocket card. A meeting was conducted with a convenient sample of 6 nurses, 3 physicians, 1 respiratory therapist (RT), and 1 pharmacist (N=11) from the 30-bed, COVID-19–designated medical ICU who volunteered to provide feedback on the new protocol. The feedback targeted the clarity and validity of the information on the protocol and pocket card, ease of use based on the card and font sizes, and delineation of roles inside and outside of the room.

The feedback from the multidisciplinary team was incorporated, and the guideline and pocket card were revised. Multimedia Appendix 1 highlights the changes incorporated into our COVID-19 CPR process based on the AHA COVID-19 Resuscitation Guideline in comparison to our standard CPR algorithm prior to COVID-19 [1]. The new protocol focused on team organization as in-room and out-of-room teams, roles and responsibilities, equipment placement, communication and coordination, the process of conducting CPR, and emphasized personal protective equipment (PPE) donning. It is worth noting that the AHA COVID-19 Resuscitation Guideline “recommended” the use of mechanical chest compression. Although these devices are used now in ICUs, their use was limited to the emergency department at the time of the study and due to a lack of resources.

The new COVID-19 CPR protocol was disseminated to all units in the hospital in the form of a pocket card by the nurse educators of the units and published in the COVID-19 resources file on the intranet. The chairman of the Resuscitation Services Committee also shared it electronically with hospital physicians.

### In Situ Simulation

We conducted 4 in situ simulation sessions to train 4 code blue teams on our COVID-19 CPR protocol and test and streamline the process outlined in the protocol. The 4 in situ simulation sessions were conducted in the medical ICU using a high-fidelity mannequin simulator (Laerdal Medical). The standard code blue scenario used for this purpose was as follow: “Patient was admitted with COVID-19. Upon entering the patient’s room, the nurse finds the patient unresponsive to verbal cues. Patient state 1: ventricular fibrillation. Patient state 2: asystole. Patient state 3: post intubation and return of spontaneous circulation.” A total of 46 health care team members—20 nurses, 15 physicians, 5 RTs, 2 anesthesiologists, 1 technician, and 3 pharmacists—participated in the 4 mock codes.

The code blue teams were aware of the study but unaware of the timing of the mock code until they heard the overhead announcement to elicit a real-life response. The Directors and Patient Care Coordinators of all units in the hospital assigned roles and responsibilities to code blue teams at the beginning of each shift and provided nurses with a copy of the guide and pocket card in advance of the simulations. Code blue procedures were announced per the current protocol (ie, no designation for a positive or suspected COVID-19 patient was provided). Upon arrival, participants were prebriefed on (1) the COVID-19 CPR protocol using the pocket card, (2) the use of a high-fidelity mannequin simulator, and (3) the focus of the simulation. Participants were encouraged to ask questions. The nurse educator (JH) and 2 intensivists (CD and ES) served as content experts for all simulation sessions. Debriefing was conducted after each session by the nurse educator who facilitated the simulation. The debriefing also solicited the participants’ feedback about the new protocol and the value of the pocket card on streamlining the resuscitation process.

Our focus during the simulation was on the changes incorporated into the resuscitation process. We observed role clarity; the effectiveness of communication between the in-room and out-of-room teams; team dynamics; appropriate PPE donning and doffing; and the availability of essential medications, supplies, and equipment to ensure that high-quality CPR was provided. We additionally observed each member’s competence in completing their designated tasks.

### Ethical Considerations

The study was approved by the Institutional Review Board of University of Texas Health (HSC20200657N).

## Results

The main issues identified in each simulation session (Multimedia Appendix 2) were debriefed to the code blue teams and used to further revise the protocol and streamline the workflow.

### First Simulation

Limiting the number of team members inside the room was one of the changes to the workflow after COVID-19 (Multimedia Appendix 1). A major issue faced in the first simulation session was the experience of fatigue by the technician from the continuous compressions. To solve this problem in subsequent simulations, we decided to place the technician with the out-of-room team, assign the compression task to the first response nurse, and add a third nurse to the in-room team to provide high-quality compressions. This change would result in 3 nurses inside the room and 2 technicians outside the room. All 3 nurses in the room could administer medications and rotate providing high-quality compressions. The technicians would serve as runners and gatekeepers to obtain equipment and ensure the proper doffing of PPE and cleaning of equipment.

Another change we implemented to the workflow was placing all first-line code medications in the Pyxis crash cart. Bringing medications from the Pyxis crash cart into the isolation room caused major workflow disruption and delayed medication administration (Multimedia Appendix 2). A viable solution was the creation of an isolation code medication package by the pharmacy. In the debriefing and subsequent mock codes, the teams were instructed to take the isolation code medication package found in a tray in the code cart into the isolation room upon initial entry, as opposed to the full tray of code medications from the crash cart or the code cart itself.

### Second Simulation

The addition of a third nurse to the in-room team allowed for high-quality compressions to be maintained and additional tasks such as medication administration and rhythm analysis to be completed by all members of the in-room team. Failure of communication between the in-room and out-of-room teams was the main issue faced in the second simulation. After COVID-19, we decided to use Cisco phones (Cisco 8821) as a communication tool between the in-room and out-of-room teams. Cisco phones are used by nurses in our hospital for daily communication with the multidisciplinary team and family members and to answer the call light. As described in Multimedia Appendix 2, Cisco phones were not the best method of communication during the mock code.

During the same day and using the same code blue team members, we decided to repeat the second simulation (Multimedia Appendix 2, Simulation 2 Part B) using the call light system (Rauland Responder 5), which also revealed communication issues (Multimedia Appendix 2). As a solution, the code blue team suggested the use of two-way radios, which were tested in the third simulation.

### Third Simulation

The two-way radios (Motorola VL50) were a better method of communication than Cisco phones and the call light system. One of the radios was assigned to one of the in-room nurses. However, due to the multiple responsibilities of the nurses on the team, the radio was sometimes left unattended. As a result, we decided to assign the main communication role in subsequent simulations to the physician team leader to narrate the code events to the recorder who was a member of the out-of-room team.

Another issue revealed in this session was a communication barrier related to wearing a respirator mask by the physician leader, which made it difficult to hear team members in close proximity and hampered communication via the two-way radios with the recorder from outside the room. The use of N-95 masks with eye goggles, as opposed to respirator masks, was suggested to enhance communication between the physician team leader and the out-of-room team in subsequent simulation sessions.

**Figure 1:**
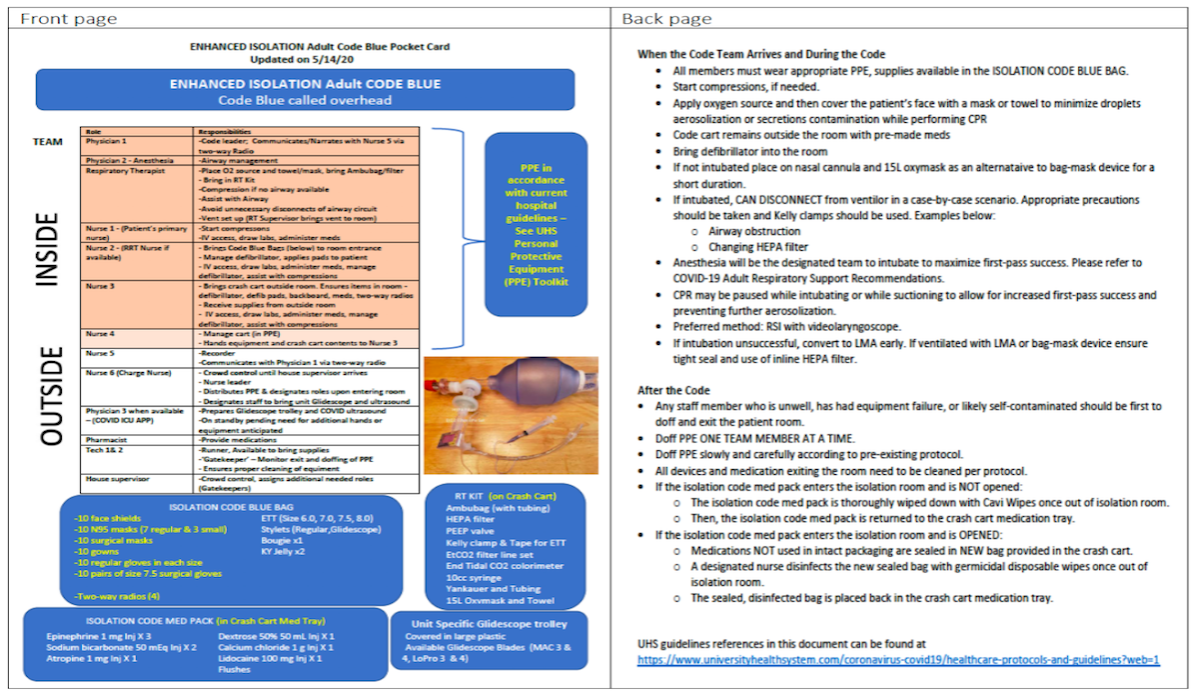
Final Pocket Card. ETT: endotracheal tube; CPR: cardiopulmonary resuscitation; ICU: intensive care unit; LMA: laryngeal mask airway; RT: respiratory therapy.

The third problem faced in this simulation was related to the commonly used supplies not being readily available in the room since the crash cart was no longer used inside the room. A respiratory therapy kit was developed and placed on each crash cart for the first responder to bring into the room along with the isolation code medication package as a solution to the problem (Multimedia Appendix 2). The list of supplies is available in Figure 1 (Multimedia Appendix 3).

### Fourth Simulation

Assigning the main communication role to the physician team leader provided effective communication between the in-room and out-of-room teams. The recorder nurse from the out-of-room team served as the communicator of the needs for the in-room team, recorded events, and directed information from outside the room (eg, “Anesthesia is here”). As a result, two-way radios were issued to each unit and placed on each crash cart in the hospital. The use of N-95 masks with eye goggles, as opposed to respirator mask, by the physician team leader allowed clear communication between the in-room and out-of-room teams. No further major issues with equipment, supplies, communication, or workflow were faced in the fourth simulation session.

### Final Protocol After Simulation

**Figure 2:**
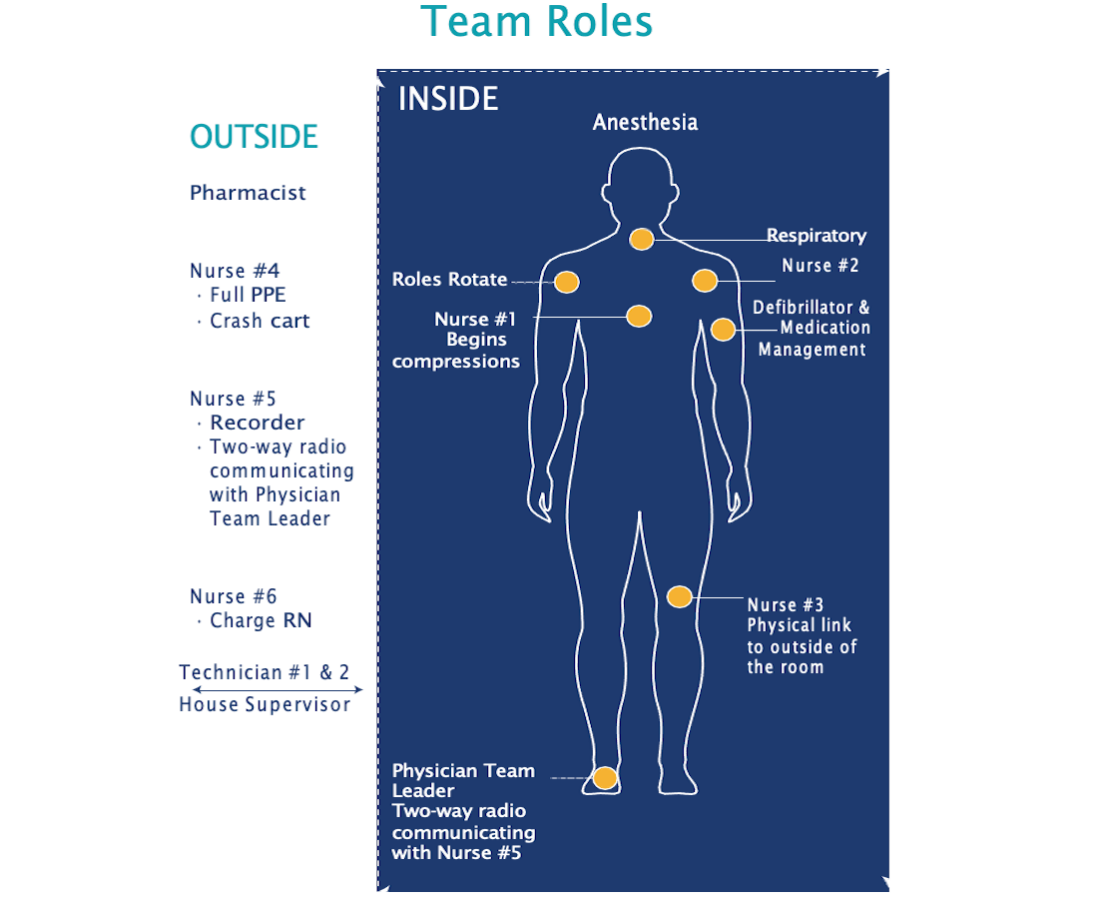
Placement of code blue team members based on the new resuscitation guide. PPE: personal protective equipment; RN: registered nurse.

The issues faced in the 4 in situ simulation sessions helped us further revise our COVID-19 CPR protocol. The changes described in Multimedia Appendix 2 (last row) were incorporated into the protocol and pocket card in its final format (Figure 1, Multimedia Appendix 3). The final placement of team members as in-room and out-of-room teams is presented in Figure 2.

### Main Debriefing Points and Lessons Learned

The debriefing sessions focused on the difference between COVID-19 code blue response (ie, infection control, role clarity, team organization, communication, and equipment) in comparison to the traditional response. Our debriefing also included a skill-related discussion when we observed a need for improvement. The main debriefing points communicated to the team and lessons learned are presented in Multimedia Appendix 4. Staff perception about the new pocket cards and protocol was also solicited during the debriefing. Staff believed that the pocket card was an easy-to-use tool to remind members of their roles and placement, the necessary equipment, and the conduct of CPR.

## Discussion

### Principal Findings

COVID-19 created an urgent need for new resuscitation policies to manage the risk of disease transmission and optimize timely intervention for the patient. The pandemic has also increased awareness of the potential for future contagion that may require modification of resuscitation policies based on the associated risks of the particular pathogen. Our in situ simulation revealed challenges unique to COVID-19 while implementing the AHA COVID-19 Resuscitation Guideline. The main challenges were related to communication, roles and responsibilities, and skills.

COVID-19 introduced unique barriers to communication during EI-CPR–related to code blue team separation into in-room and out-of-room teams; the need to maintain a closed door between the teams to contain aerosol-generating procedures that hindered the visibility of the procedure and communication clues; and the need for donning PPE and protective measures. Two-way radios were an effective communication tool between the in-room and out-of-room teams. However, communication via two-way radios is not as intuitive as face-to-face communication. Although easy to use, two-way radios allow half-duplex communication where only one person can talk at a time and hinder the natural flow of communication. Additionally, the radios are handheld and require a push on the talk button for use, which limits the clinicians’ physical movement in emergency procedures such as CPR. Furthermore, the radios need a closed loop of communication (such as “roger that”). Practicing communication using the radios and ensuring appropriate functioning and charge of the devices are important to minimize communication problems.

The few available studies on COVID-19–related CPR guidelines used other communication methods between the in-room and out-of-room teams. For example, Foong et al [5] used a whiteboard. Although cost-effective, a whiteboard is limited to visual communication and requires team members to keep looking at the board to read the written messages while performing time-sensitive procedures. Cheruku et al [6] used hospital-based mobile phones and networked videoconferencing, whereas Silver et al [7] used baby monitors. The 3 studies indicated the success of these communication strategies. Baby monitors are a cost-effective solution and have been used in Canadian hospitals to facilitate communication during CPR [10].

The negative effects of N-95 masks and face shields on speech perception among health care workers in the COID-19 pandemic were recently examined by Bandaru et al [11]. The authors found a significant increase in speech reception threshold and a significant decrease in speech discrimination scores, suggesting the use of alternate methods of communication. Despite the challenges faced by health care practices worldwide, COVID-19 may open opportunities for communication companies to develop intuitive, hand-free, full-duplex communication systems.

The organization of team members into in-room and out-of-room teams depends on the number of team members, available resources, and system policies. In our setting, it was ideal to have 2 physicians, 3 nurses, and an RT in the room. Our out-of-room team consisted of 2 nurses, 2 technicians, a pharmacist, a gatekeeper, and another physician if needed. Our teams’ organization differed slightly from Cheruku et al [6], who included only 1 in-room physician and based their practice on remote medication delivery and adjustment of the ventilator and equipment [6]. Similar to our practice, 2 physicians in the room were used by Foong et al [5]. However, airway management was a responsibility of one of the physicians and an RT was not part of the resuscitation team in that study [5]. Despite the slight differences among studies in team organization, preassignment of roles to team members was emphasized to decrease role confusion and facilitate an effective response to a COVID-19 code blue emergency.

Similar to Cheruku et al [6], the lack of infection control was not an issue revealed in our study due to the fact that our staff was extensively trained on PPE donning and doffing. On the other hand, Foong et al [5] reported violations in infection control measures related to PPE donning and doffing, cross contamination between the in-room and out-of-room teams, and failure to comply with hand hygiene standards.

### Limitations

Our study shed light on the challenges to implement the AHA COVID-19 Resuscitation Guideline. Our results are likely generalizable to other situations in which enhanced precautions are necessary. The 4 in situ simulation sessions identified and tested solutions to these challenges. Hospital systems need to implement new CPR algorithms to minimize the spread of COVID-19 [2]. The findings of this study should be interpreted in light of the following limitations. First, the study was conducted in a high-tech, Level I trauma magnet facility and well-equipped ICUs with sufficient resources. Additionally, our system provides robust PPE donning and doffing training programs. Therefore, the process of CPR and distribution and responsibilities of the in-room and out-of-room teams may not be generalizable to settings with limited resources. Second, the main focus of this study was identifying challenges and testing solutions to the new COVID-19 CPR protocol disseminated via a pocket card that includes team response to COVID-19–related EI-CPR. Our study did not focus on team competence on CPR, resuscitation metrics (eg, time to successful intubation and the quality of compression), effective handoff from the first responder nurse to the code blue team, or time taken for code blue. The study also did not use tools such as Simulation Team Assessment, and the results were solely based on observation and the debriefing process. Expanding this study in the future to include these outcome measures and tools is warranted. Third, team response during simulation may not reflect the actual performance in real cardiac arrest situations. Team members may lack the motivation to perform well during simulations or may overperform due to the Hawthorne effect. This suggests the need to observe performance during actual cardiac arrest situations.

### Conclusions

COVID-19 introduced unique challenges to implementing the AHA COVID-19 Resuscitation Guideline. The main challenges were related to communication, roles and responsibilities, and equipment placement. The new COVID-19 CPR protocol disseminated via a pocket card provided an easy-to-use tool for code blue teams to remember their placement, carry out their roles and responsibilities, interact effectively, and place equipment properly. Preassignment of roles to team members is crucial to decrease role confusion and facilitate effective response. The two-way radios were effective to facilitate communication during code blue events.

## Appendix

Multimedia Appendix 1 Difference in code blue process between the standard cardiopulmonary resuscitation (CPR) algorithm and the AHA (American Heart Association) COVID-19–related Interim Resuscitation Guideline.

Multimedia Appendix 2 Process of revising COVID-19 cardiopulmonary resuscitation (CPR) protocol.

Multimedia Appendix 3 Main debriefing points communicated to the team and lessons learned.

Multimedia Appendix 4 Final pocket card.

## Abbreviations

AHA: American Heart Association
CPR: cardiopulmonary resuscitation
EI-CPR: enhanced-isolation cardiopulmonary resuscitation
ICU: intensive care unit
PPE: personal protective equipment
RT: respiratory therapist

## References

[1] Callaway Clifton W, Soar Jasmeet, Aibiki Mayuki, Böttiger Bernd W, Brooks Steven C, Deakin Charles D, Donnino Michael W, Drajer Saul, Kloeck Walter, Morley Peter T, Morrison Laurie J, Neumar Robert W, Nicholson Tonia C, Nolan Jerry P, Okada Kazuo, O'Neil Brian J, Paiva Edison F, Parr Michael J, Wang Tzong-Luen, Witt Jonathan, Advanced Life Support Chapter Collaborators (2015). Part 4: advanced life support: 2015 international consensus on cardiopulmonary resuscitation and emergency cardiovascular care science with treatment recommendations. Circulation.

[2] Edelson Dana P, Sasson C, Chan Paul S, Atkins Dianne L, Aziz Khalid, Becker Lance B, Berg Robert A, Bradley SM, Brooks SC, Cheng Adam, Escobedo Marilyn, Flores Gustavo E, Girotra Saket, Hsu Antony, Kamath-Rayne Beena D, Lee Henry C, Lehotsky Rebecca E, Mancini Mary E, Merchant Raina M, Nadkarni Vinay M, Panchal Ashish R, Peberdy Mary Ann R, Raymond Tia T, Walsh Brian, Wang David S, Zelop Carolyn M, Topjian Alexis A, American Heart Association ECC Interim COVID Guidance Authors (2020). Interim guidance for basic and advanced life support in adults, children, and neonates with suspected or confirmed COVID-19: from the emergency cardiovascular care committee and get with the guidelines-resuscitation adult and pediatric task forces of the American Heart Association. Circulation.

[3] Nolan JP, Monsieurs K G, Bossaert L, Böttiger B W, Greif R, Lott C, Madar J, Olasveengen T M, Roehr C C, Semeraro F, Soar J, Van de Voorde P, Zideman D A, Perkins G D, European Resuscitation Council COVID-Guideline Writing Groups (2020). European Resuscitation Council COVID-19 guidelines executive summary. Resuscitation.

[4] Craig S, Cubitt M, Jaison A, Troupakis Steven, Hood Natalie, Fong Christina, Bilgrami Adnan, Leman Peter, Ascencio-Lane Juan Carlos, Nagaraj Guruprasad, Bonning John, Blecher Gabriel, Mitchell Rob, Burkett Ellen, McCarthy Sally M, Rojek Amanda M, Hansen Kim, Psihogios Helen, Allely Peter, Judkins Simon, Foong Lai Heng, Bernard Stephen, Cameron Peter A (2020). Management of adult cardiac arrest in the COVID-19 era: consensus statement from the Australasian College for Emergency Medicine. Med J Aust.

[5] Foong TW, Hui Ng ES, Wee Khoo CY, Ashokka B, Khoo D, Agrawal R (2020). Rapid training of healthcare staff for protected cardiopulmonary resuscitation in the COVID-19 pandemic. Br J Anaesth.

[6] Cheruku S, Dave S, Goff K, Park C, Ebeling C, Cohen L, Styrvoky K, Choi C, Anand V, Kershaw C (2020). Cardiopulmonary resuscitation in intensive care unit patients with coronavirus disease 2019. J Cardiothorac Vasc Anesth.

[7] Silver S, Amaral N, Heng D, Mundle W (2020). Simulation-based learning during COVID-19: a teaching strategy for protected code blues. J Contin Educ Nurs.

[8] Sharara-Chami R, Lakissian Z, Farha R, Tamim H, Batley N (2020). In-situ simulation for enhancing teamwork in the emergency department. Am J Emerg Med.

[9] Yager P, Collins C, Blais C, O'Connor K, Donovan P, Martinez M, Cummings B, Hartnick C, Noviski N (2016). Quality improvement utilizing in-situ simulation for a dual-hospital pediatric code response team. Int J Pediatr Otorhinolaryngol.

[10] Ward M St Joe's turns to baby monitors to communicate during COVID-19 pandemic. CBC News.

[11] Bandaru SV, Augustine AM, Lepcha A, Sebastian S, Gowri M, Philip A, Mammen MD (2020). The effects of N95 mask and face shield on speech perception among healthcare workers in the coronavirus disease 2019 pandemic scenario. J Laryngol Otol.

