# Supplementary material for: Developing and Testing a Protocol for Managing Cardiopulmonary Resuscitation of Patients with Suspected or Confirmed COVID-19: In Situ Simulation Study
Source: JMIR Nurs. 2022 Jun 16;5(1):e38044. doi: 10.2196/38044 (PMC9205423; doi:10.2196/38044)
Supplement: Multimedia Appendix 1 [file nursing_v5i1e38044_app1.docx]

**Multimedia Appendix 1.** Difference in code blue process between the standard cardiopulmonary resuscitation (CPR) algorithm and the AHA (American Heart Association) COVID-19-Related Interim Resuscitation Guideline

| **Area** | **CPR process**  **Based on AHA standard CPR algorithm (Prior COVID-19)** | **Enhanced-Precautions CPR Process based on AHA COVID-19-Related Interim Resuscitation Guideline** **(Post COVID-19)** |
| --- | --- | --- |
| Placement of team members and roles and  responsibilities | All team members are inside the room.   - Nurse 1: Primary nurse, provides information about the patient, next to physician team leader - Nurse 2: Medication/defibrillator nurse, next to crash cart. - Nurse 3: Recorder, next to physician team leader - Nurse 4: Crowd control, minimizes participants, (mobile, no designated place to stand) - Nurse 5: Nurse team leader, makes sure all roles are filled, helps secure a higher-level bed if needed and communicates that with the house supervisor (mobile, no designated place to stand) - Respiratory Therapist (RT): Airway role, head of the bed - Technician 1 and 2: Provide “continuous” compressions, on either side of the bed - Physician 1: Physician team leader, foot of the bed - Physician 2: Airway management, head of the bed - Pharmacist: Manages medication, near crash cart | Team is divided into in-room and out-of-room teams.  In-room Team:   - Nurse 1: intravenous access, draws labs, administers medications, manages defibrillator, brings isolation code blue bag (has additional PPE or personal protected equipment and airway supplies) off of the crash cart upon entry to the room, stands on one side of the bed but moves based on needs - Nurse 2: Assists Nurse 1, communicates with Nurse 3 (out of room) - Physician 1: ICU-code leader, brings isolation code blue bag (if not brought by Nurse 1), foot of the bed - Physician 2 Anesthesia: Airway management, brings airway bag, head of the bed - RT: Assists with airway, ventilator set up and connectivity, head of the bed - Technician: Compressions (at a rate of 30 compressions followed by 2 breaths), side of the bed   Out-of-room team**:**   - Nurse 3: Communicates with Nurse 2, hands equipment and medications to Nurse 2 - Nurse 4: Manages cart, right outside the door in full PPE - Nurse 5: Recorder, stands by the window. - Nurse 6: Gatekeeper, provides crowd control via overseeing access to room, near the window or glass door - Pharmacist: Provides medications, stands by crash cart with Nurse 4 - Technician: Prioritize donning PPE, available to bring supplies, brings GlideScope from the supply room, stands near Nurse 4 - House supervisor: Crowd control, assigns additional roles if needed, mobile |
| Equipment | - All first-line code medications are in crash cart - Crash cart inside the room with all available supplies | - All first-line code medications are in the pyxis - Crash cart outside the room - Isolation code blue bag that has additional PPE and airway supplies |
|  | - PPE depends on the patient diagnosis, readily available PPE in the cabinets outside the patient’s room - N-95 masks availability was not an issue before COVID-19 | - The availability of gowns and gloves remained the same - N95 masks were not readily available in these cabinets after the onset of COVID. N95 masks were issued to staff by management to preserve the supply - Eye protection was required for staff working with COVID-19 patients - Eye wear required signs posted on doors |
| Communication | - Verbal, intuitive among responders in the room | - Cisco phones |
| Infection control | - No gatekeeper - Prioritize donning and doffing - One team - Standard precautions | - Donning and doffing of PPE was observed by the gatekeeper - Two teams - Gloves were sanitized prior to removal - Posters made by the Centers for Disease Control and Prevention or CDC for the proper steps in donning and doffing were posted in rooms - Use a towel to cover the patient’s mouth and nose to prevent potential spread of the virus until intubation |
