# Supplementary material for: Developing and Testing a Protocol for Managing Cardiopulmonary Resuscitation of Patients with Suspected or Confirmed COVID-19: In Situ Simulation Study
Source: JMIR Nurs. 2022 Jun 16;5(1):e38044. doi: 10.2196/38044 (PMC9205423; doi:10.2196/38044)
Supplement: Multimedia Appendix 2 [file nursing_v5i1e38044_app2.docx]

**Multimedia Appendix 2**. Process of revising COVID-19 CPR (cardiopulmonary resuscitation) protocol

| **Phase** | **Issues** | **Solution** | **Comments** |
| --- | --- | --- | --- |
| **First Simulation** | Technician experienced fatigue from continuous compressions. | - - - - Replace technician with a nurse after 2-3 minutes at a rate of 100-120/min.       - Respiratory therapist to assist with compressions when not assisting with airway. | For subsequent simulations, place the technician with the out-of-room team, assign compression to the first response nurse, and add a third nurse to the in-room team to provide high-quality compressions.  By this solution, the three nurse responders in the room will be responsible for the following tasks:   - - - - Administer medications       - Apply defibrillator, analyze rhythm, deliver shock       - Compressions       - Obtain intravenous access       - Obtain supplies from outside the room       - Placing lines and tubes, drawing blood |
|  | First-line code medications were kept in Pyxis to prevent contamination of medications in crash cart. This resulted in:   - - - - Different places to get medications from (crash cart and Pyxis) and a delay in preparing and administering medications given the fact that there were only two Pyxis in each unit and that badge access to medication room is granted to nurses from the unit.       - Using the medications as needed in the crash cart resulted in the door staying open, which may increase the chance of contamination. | - - - - Development of an isolation code medication package by pharmacy. | - - - - For the list of supplies in the isolation code blue medication package, please see Figure 3.       - Further medications needed can be retrieved and handed into the anteroom by a nurse or a pharmacist.       - The medication package can also be used in non-COVID-19/non-isolation rooms. |
| **Second Simulation Part A** | Failure of communication between the in-room and out-of-room teams. | - - - - Use Cisco phone. | - - - - It was too hard to understand the communicated messages and to use Cisco phones with a face shield and N95 masks.       - Cisco phones are not as readily available on crash cart.       - Nurses have to give the physician team leader their phones and call the Cisco phone of the recorder outside of the room.       - The physician could not call the nurse from out-of-room team because they did not know the number to call for emergency response. |
| **Second Simulation Part B** | Failure of communication between the in-room and out-of-room teams. | - - - - Use the call light system.       - The in-room team send their messages to out-of-room team who receive the message via a speaker. | - - - - Noise in the room was a major factor for interrupted and miscommunication. |
| **Third Simulation** | Failure of communication between the in-room and out-of-room teams. | - - - - Use two-way radios by nurses from the in-room and out-of-room teams. | - - - - The radio was sometimes left unattended. This resulted in not communicating all steps of the code to the recorder nurse from the out-of-room team.       - The need to assign communication to the physician leader from the in-room team was identified. |
|  | Wearing respirator mask is a communication barrier. | - - - - Use of N-95 with eye goggles. | - |
|  | Commonly used supplies were not readily available in the room since crash cart is not going inside the room. | - - - - Respiratory therapy (RT) kit was developed and placed on each crash cart. | RT kit provided immediate access to key supplies:   - - - - Viral filter for the ambubag (Hepa filter)       - Peep valve       - Kelly clamp       - End-tidal Carbone dioxide or EtCO2 filter line set       - EtCo2 detector       - Tape for ETT (endotracheal tube), 10 cc syringe       - Yankauer and tubing       - Passive way to provide oxygen (Oxymask)       - Sterile towel to cover the face during compressions |
| **Fourth Simulation** | Failure of communication between the in-room and out-of-room teams. | Assign communication to the physician leader from the in-room team. | - - - - The battery of one of the radios died toward the end of the simulation. We decided to add additional radios in the code blue isolation bag. |
| **Final Changes to Protocol and Pocket Card** | - - - - A third nurse was added to the in-room team and the technician joined the out-of-room team.       - An easily obtained isolation code medication package was created and included all first-line medications.       - Two-way radios were established as the communication tool of choice between the in-room and out-of-room teams.       - N-95 with eye goggles was used to enhance communication between the physician team leader and out-of-room team.       - A RT kit for the commonly used supplies was created and was placed on each crash cart. | | |
