# Supplementary material for: Developing and Testing a Protocol for Managing Cardiopulmonary Resuscitation of Patients with Suspected or Confirmed COVID-19: In Situ Simulation Study
Source: JMIR Nurs. 2022 Jun 16;5(1):e38044. doi: 10.2196/38044 (PMC9205423; doi:10.2196/38044)
Supplement: Multimedia Appendix 3 [file nursing_v5i1e38044_app3.docx]

Multimedia Appendix 4: Final pocket card

| Front Page | Back Page |
| --- | --- |
| **ENHANCED ISOLATION Adult CODE BLUE**  Code Blue called overhead   \| **Role** \| **Responsibilities** \| \| --- \| --- \| \| Physician 1 \| -Code leader; Communicates/Narrates with Nurse 5 via two-way Radio \| \| Physician 2 - Anesthesia \| -Airway management \| \| Respiratory Therapist \| -Place O2 source and towel/mask, bring Ambubag/filter  - Bring in RT Kit  -Compression if no airway available  -Assist with Airway  -Avoid unnecessary disconnects of airway circuit  -Vent set up (RT Supervisor brings vent to room) \| \| Nurse 1 - (Patient’s primary nurse) \| -Start compressions  -IV access, draw labs, administer meds \| \| Nurse 2 - (RRT Nurse if available) \| - Brings Code Blue Bags (below) to room entrance  - Manage defibrillator, applies pads to patient  - IV access, draw labs, administer meds, manage defibrillator, assist with compressions \| \| Nurse 3 \| - Brings crash cart outside room. Ensures items in room - defibrillator, defib pads, backboard, meds, two-way radios  - Receive supplies from outside room  - IV access, draw labs, administer meds, manage defibrillator, assist with compressions \| \| Nurse 4 \| - Manage cart (in PPE)  - Hands equipment and crash cart contents to Nurse 3 \| \| Nurse 5 \| -Recorder  -Communicates with Physician 1 via two-way radio \| \| Nurse 6 (Charge Nurse) \| - **Crowd control** until house supervisor arrives  - Nurse leader  - Distributes PPE & designates roles upon entering room  - Designates staff to bring unit Glidescope and ultrasound \| \| Physician 3 when available – (COVID ICU APP) \| -Prepares Glidescope trolley and COVID ultrasound  -On standby pending need for additional hands or equipment anticipated \| \| Pharmacist \| -Provide medications \| \| Tech 1& 2 \| -Runner, Available to bring supplies  -‘Gatekeeper’ – Monitor exit and doffing of PPE  - Ensures proper cleaning of equipment \| \| House supervisor \| -Crowd control, assigns additional needed roles (Gatekeepers) \|   **TEAM**      **PPE in accordance with current hospital guidelines – See hospital Personal Protective Equipment (PPE) Toolkit**  **INSIDE**  **OUTSIDE**  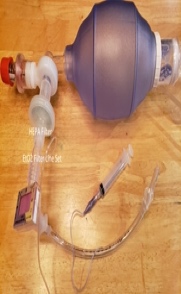  **ISOLATION CODE MED PACK (in Crash Cart Med Tray)**   \| **Epinephrine 1 mg Inj X 3** \| **Dextrose 50% 50 mL Inj X 1** \| \| --- \| --- \| \| **Sodium bicarbonate 50 mEq Inj X 2** \| **Calcium chloride 1 g Inj X 1** \| \| **Atropine 1 mg Inj X 1** \| **Lidocaine 100 mg Inj X 1** \| \| **Flushes** \|  \|   **Unit Specific Glidescope trolley**  -Covered in large plastic  -Available Glidescope Blades (MAC 3 & 4, LoPro 3 & 4)  **ISOLATION CODE BLUE BAG**   \| 10 face shiel \| Bougie x1 \| \| --- \| --- \| \| - ETT (Size 6.0, 7.0, 7.5, 8.0) \| 10 gowns \| \| Stylets (Regular, Glidescope) \| 10 regular gloves in each size \| \| 10 surgical masks \| KY Jelly x2 \| \| 10 N95 masks (7 regular & 3 small) \| Two-way radios (4) \| \| 10 pairs of size 7.5 surgical gloves \|  \|     **RT KIT- (on Crash Cart)**   - **Ambubag (with tubing** - **HEPA filter** - **PEEP valve** - **Kelly clamp & Tape for ETT** - **EtCO2 filter line set** - **End Tidal CO2 colorimeter** - **10cc syringe** - **Yankauer and Tubing** - **15L Oxymask and Towel** | **When the Code Team Arrives and During the Code**   - All members must wear appropriate PPE, supplies available in the ISOLATION CODE BLUE BAG. - Start compressions, if needed. - Apply oxygen source and then cover the patient’s face with a mask or towel to minimize droplets aerosolization or secretions contamination while performing CPR - Code cart remains outside the room with pre-made meds - Bring defibrillator into the room - If not intubated place on nasal cannula and 15L oxymask as an alternative to bag-mask device for a short duration. - If intubated, CAN DISCONNECT from ventilator in a case-by-case scenario. Appropriate precautions should be taken and Kelly clamps should be used. Examples below:   - Airway obstruction   - Changing HEPA filter - Anesthesia will be the designated team to intubate to maximize first-pass success. Please refer to COVID-19 Adult Respiratory Support Recommendations. - CPR may be paused while intubating or while suctioning to allow for increased first-pass success and preventing further aerosolization. - Preferred method: RSI with videolaryngoscope. - If intubation unsuccessful, convert to LMA early. If ventilated with LMA or bag-mask device ensure tight seal and use of inline HEPA filter.   **After the Code**   - Any staff member who is unwell, has had equipment failure, or likely self-contaminated should be first to doff and exit the patient room. - Doff PPE ONE TEAM MEMBER AT A TIME. - Doff PPE slowly and carefully according to pre-existing protocol. - All devices and medication exiting the room need to be cleaned per protocol. - If the isolation code med pack enters the isolation room and is NOT opened:   - The isolation code med pack is thoroughly wiped down with Cavi Wipes once out of isolation room.   - Then, the isolation code med pack is returned to the crash cart medication tray. - If the isolation code med pack enters the isolation room and is OPENED:   - Medications NOT used in intact packaging are sealed in NEW bag provided in the crash cart.   - A designated nurse disinfects the new sealed bag with germicidal disposable wipes once out of isolation room.   - The sealed, disinfected bag is placed back in the crash cart medication tray.   The hospital guidelines references in this document can be found at <https://www.universityhealthsystem.com/coronavirus-covid19/healthcare-protocols-and-guidelines?web=1> |
