# Supplementary material for: Developing and Testing a Protocol for Managing Cardiopulmonary Resuscitation of Patients with Suspected or Confirmed COVID-19: In Situ Simulation Study
Source: JMIR Nurs. 2022 Jun 16;5(1):e38044. doi: 10.2196/38044 (PMC9205423; doi:10.2196/38044)
Supplement: Multimedia Appendix 4 [file nursing_v5i1e38044_app4.docx]

**Multimedia Appendix 3**. Main debriefing points communicated to the team and lessons learned

| **Area** | **Main points debriefed** | **Lesson learned** |
| --- | --- | --- |
| Infection control | - The gatekeeper should allow only one person to exit at a time. - Team members need to physically distance themselves while waiting to doff and exit the room. - It is important to cover the patient face with a towel, gown, or sheet before starting compressions. | - Practicing PPE donning and doffing is essential. - The role of the gatekeeper (technicians 1 and 2) is necessary to guide clinicians on how to properly doff the PPE before exiting the room. |
| Role clarity and team placement | - The followings were emphasized: - Technician 1 and 2 to bring the unit GlideScope and ultrasound. - Physician team leader to provide feedback on the quality of compressions. - Oxygen mask and sterile towel are in the RT kit and should be brought in with the initial responders. - Nurse 4 is essential to manage the crash cart and to be gowned up in PPE to assist inside the room when needed. | - The pre-assignment of COVID-19 code blue roles into two teams at the beginning of a shift is crucial for task coordination, delegation, and effective resuscitation. - Clear roles and responsibilities and effective team dynamic are crucial to streamline the workflow and minimize errors in resuscitation. - Simulation is an effective method to identify the ideal organization and tasks of the in-room and out-of-room response teams. |
| Communication | - The two-way radios should be operated by the physician team leader inside the room and the recorder from outside the room. - Team members are to identify themselves and their roles to the team upon arrival and during the event to clear any role confusion. | - Code blue during COVID-19 introduced communication barriers. - The two-way radios is an efficient solution to establish communication during a high-stakes event. - Communication via two-way radios is not intuitive and practice is necessary to prevent problems. - Checking the batteries of the radios before the start of each shift is necessary to eliminate communication delay. - The use of N-95 with eye goggles enhanced the communication of the physician team leader. |
| Skills | - During the debriefing, we also familiarized the staff with the skill portion of code blue response. These include, - How to put the bed in cardiopulmonary resuscitation or CPR mode. - Review high-quality compressions. - How to remove the headboard for airway management. - Medication assembly. - How to ensure suction is readily available. - EtCo2 or end tidal carbon dioxide level monitoring on defibrillator. - Defibrillator function, including attaching pads. | - A strong foundation for code blue response is necessary due to less staff being available in the room and the more structured nature of the roles during a COVID-19 code blue response. - Staff responding to a code blue event should familiarize themselves with the protocol including the supplies available to them to bring to the room. - An experienced nurse is the best option for the role of nurse 5- recorder. This role requires recording the timing of the code, documenting code events, and constant communication with the in-room team. |
